# The genome editing revolution: review

**DOI:** 10.1186/s43141-020-00078-y

**Published:** 2020-10-29

**Authors:** Ahmad M. Khalil

**Affiliations:** Department of Biological Sciences, Yarmouk University, Irbid, Jordan

**Keywords:** CRISPR-Cas system, Gene editing, Gene therapy, Genome editing, Meganucleases, TALEN, ZFN

## Abstract

**Background:**

Development of efficient strategies has always been one of the great perspectives for biotechnologists. During the last decade, genome editing of different organisms has been a fast advancing field and therefore has received a lot of attention from various researchers comprehensively reviewing latest achievements and offering opinions on future directions. This review presents a brief history, basic principles, advantages and disadvantages, as well as various aspects of each genome editing technology including the modes, applications, and challenges that face delivery of gene editing components.

**Main body:**

Genetic modification techniques cover a wide range of studies, including the generation of transgenic animals, functional analysis of genes, model development for diseases, or drug development. The delivery of certain proteins such as monoclonal antibodies, enzymes, and growth hormones has been suffering from several obstacles because of their large size. These difficulties encouraged scientists to explore alternative approaches, leading to the progress in gene editing. The distinguished efforts and enormous experimentation have now been able to introduce methodologies that can change the genetic constitution of the living cell. The genome editing strategies have evolved during the last three decades, and nowadays, four types of “programmable” nucleases are available in this field: meganucleases, zinc finger nucleases, transcription activator-like effector nucleases, and the clustered regularly interspaced short palindromic repeats (CRISPR)/CRISPR associated protein 9 (Cas9) (CRISPR/Cas-9) system. Each group has its own characteristics necessary for researchers to select the most suitable method for gene editing tool for a range of applications. Genome engineering/editing technology will revolutionize the creation of precisely manipulated genomes of cells or organisms in order to modify a specific characteristic. Of the potential applications are those in human health and agriculture. Introducing constructs into target cells or organisms is the key step in genome engineering.

**Conclusions:**

Despite the success already achieved, the genome editing techniques are still suffering certain difficulties. Challenges must be overcome before the full potential of genome editing can be realized.

## Background

In classical genetics, the gene-modifying activities were carried out selecting genetic sites related to the breeder’s goal. Subsequently, scientists used radiation and chemical mutagens to increase the probability of genetic mutations in experimental organisms. Although these methods were useful, they were time-consuming and expensive. Contrary to this, reverse genetics goes in the opposite direction of the so-called forward genetic screens of classical genetics. Reverse genetics is a method in molecular genetics that is used to help understanding the function of a gene by analyzing the phenotypic effects of specific engineered gene sequences. Robb et al. [68] defined and compared the three terms: “genome engineering”, “genome editing”, and “gene editing”. Genome engineering is the field in which the sequence of genomic DNA is designed and modified. Genome editing and gene editing are techniques for genome engineering that incorporate site-specific modifications into genomic DNA using DNA repair mechanisms. Gene editing differs from genome editing by dealing with only one gene.

This review briefly presents the evolution of genome editing technology over the past three decades using PubMed searches with each keyword of genome-editing techniques regarding the brief history, basic principles, advantages and disadvantages, as well as various aspects of each genome editing technology including the modes, future perspective, applications, and challenges.

## Main text

Genome-wide editing is not a new field, and in fact, research in this field has been active since the 1970s. The real history of this technology started with pioneers in genome engineering [36, 59]. The first important step in gene editing was achieved when researchers demonstrated that when a segment of DNA including homologous arms at both ends is introduced into the cell, it can be integrated into the host genome through homologous recombination (HR) and can dictate wanted changes in the cell [10]. Employing HR alone in genetic modification posed many problems and limitations including inefficient integration of external DNA and random incorporation in undesired genomic location. Consequently, the number of cells with modified genome was low and uneasy to locate among millions of cells. Evidently, it was necessary to develop a procedure by which scientists can promote output. Out of these limitations, a breakthrough came when it was figured out that, in eukaryotic cells, more efficient and accurate gene targeting mechanisms could be attained by the induction of a double stranded break (DSB) at a specified genomic target [70].

**Fig. 1:**
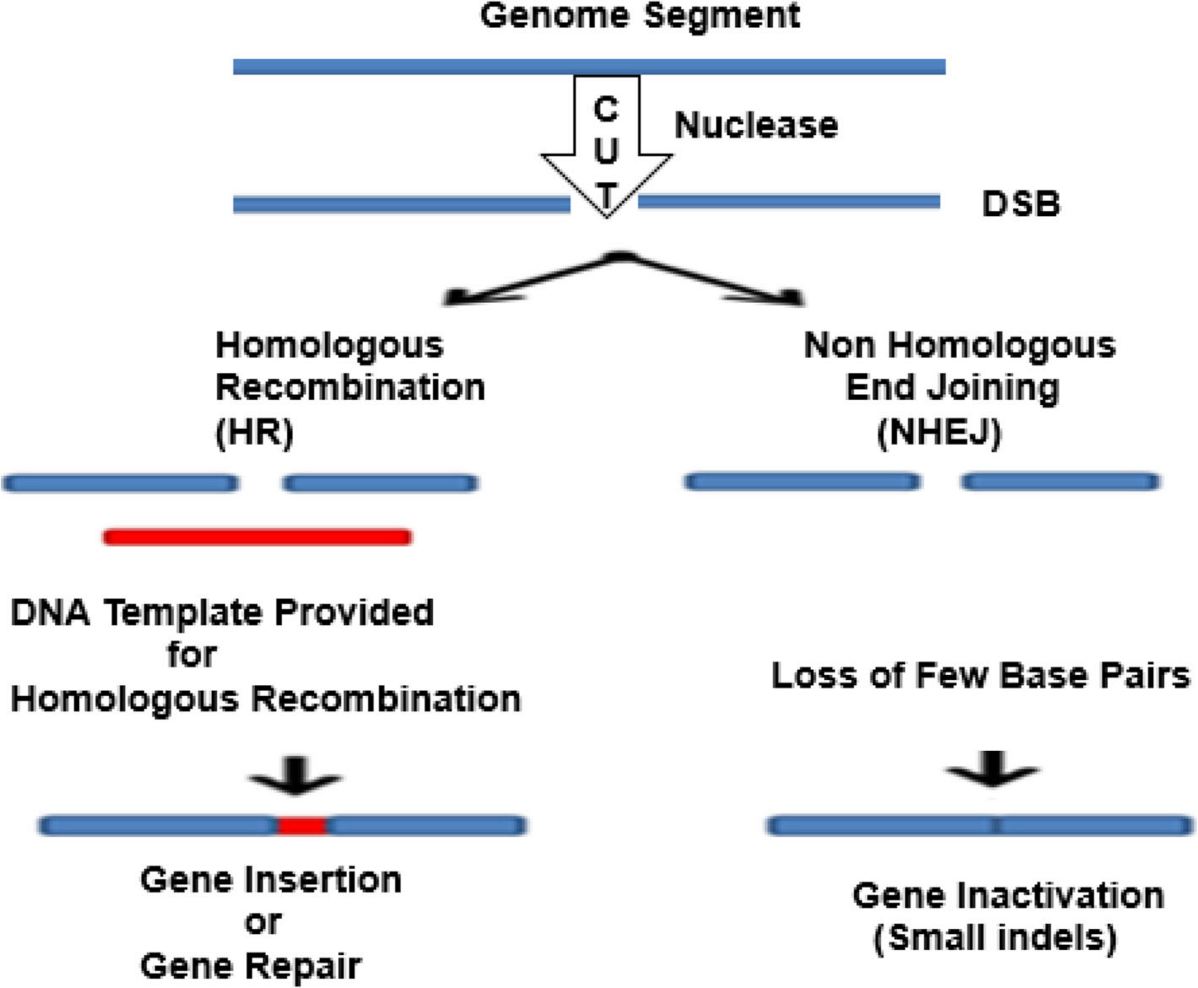
Genome editing outcomes. Genome editing nucleases induce double-strand breaks (DSBs). The breaks are repaired through two ways: by non-homologous end joining (NHEJ) in the absence of a donor template or via homologous recombination (HR) in the presence of a donor template. The NHEJ creates few base insertions or deletion, resulting in an indel, or in frameshift that causes gene disruption. In the HR pathway, a donor DNA (a plasmid or single-stranded oligonucleotide) can be integrated to the target site to modify the gene, introducing the nucleotides and leading to insertion of cDNA or frameshifts induction. (Adapted from [78])

Furthermore, scientists found that if an artificial DNA restriction enzyme is inserted into the cell, it cuts the DNA at specific recognition sites of double-stranded DNA (dsDNA) sequences. Thus, both the HR and non-homologous end joining (NHEJ) repair can be enhanced [14]. Various gene editing techniques have focused on the development and the use of different endonuclease-based mechanisms to create these breaks with high precision procedures [53, 78] (Fig. 1). The mode of action of what is known as site-directed nucleases is based on the site-specific cleavage of the DNA by means of nuclease and the triggering of the cell’s DNA repair mechanisms: HR and NHEJ.

One of the limitations in this procedure is that it has to be activated only in proliferating cells, adding that the level of activity depends on cell type and target gene locus [72]. Tailoring of repair templates for correction or insertion steps will be affected by these differences. Several investigations have determined ideal homology-directed repair (HDR) donor configurations for specific applications in specific models systems [67]. The differences in the activities of the DNA repair mechanisms will also influence the efficiency of causing indel mutations through NHEJ or the classical microhomology-mediated end joining (c-MMEJ) pathway, and even the survival of the targeted cells. The production of such repair in the cell is a sign of a characteristic that errors may occur during splicing the ends and cause the insertion or deletion of a short chain. Simply speaking, gene editing tools involve programmed insertion, deletion, or replacement of a specific segment of in the genome of a living cell. Potential targets of gene editing include repair of mutated gene, replacement of missing gene, interference with gene expression, or overexpression of a normal gene.

The human genome developments paved the way to more extensive use of the reverse genetic analysis technique. Nowadays, two methods of gene editing exist: one is called “targeted gene replacement” to produce a local change in an existing gene sequence, usually without causing mutations. The other one involves more extensive changes in the natural genome of species in a subtler way.

**Fig. 2:**
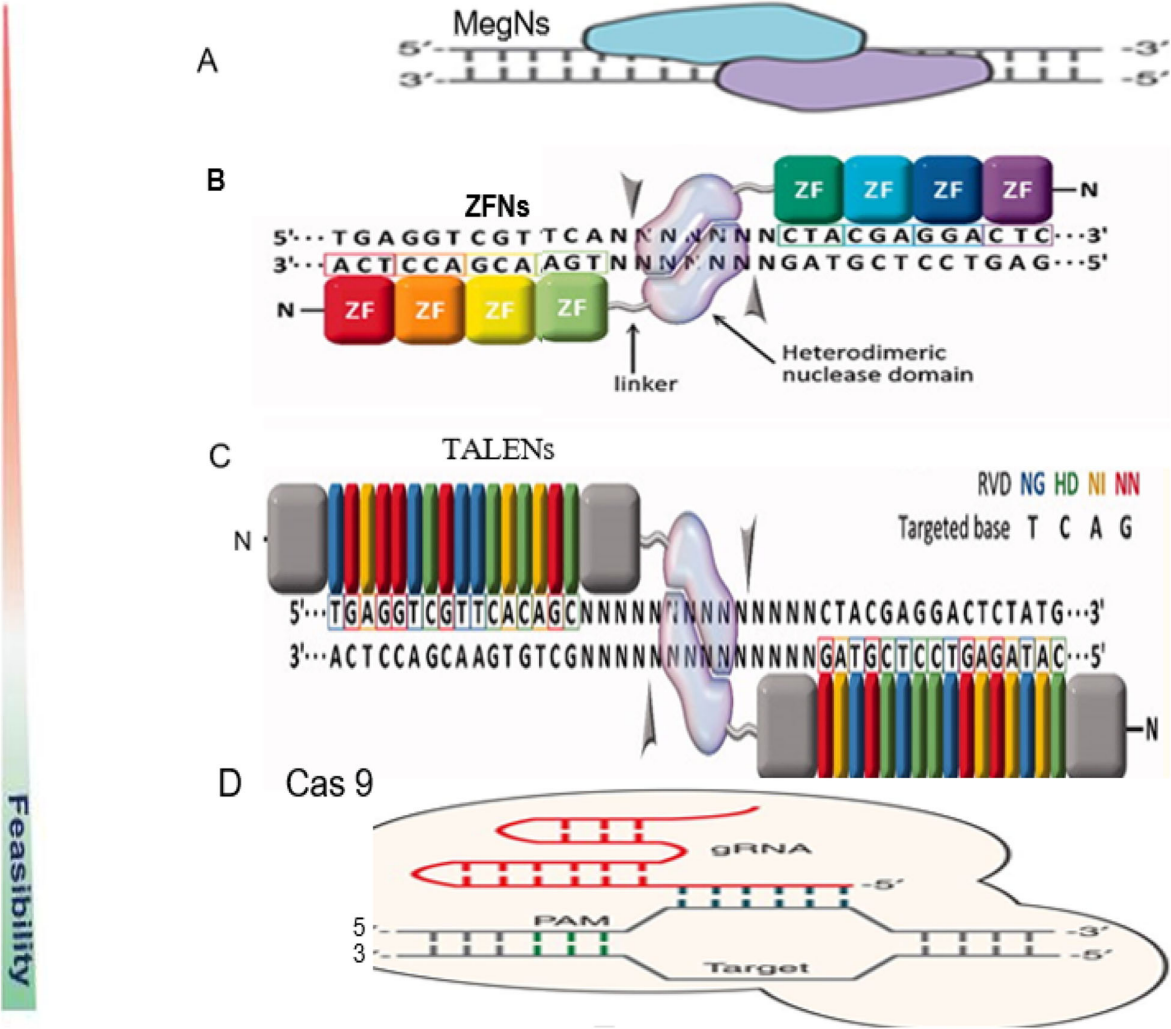
Schematic diagram of the four endonucleases used in gene editing technologies. **a** Meganuclease (MegN) that generally cleaves its DNA substrate as a homodimer. **b** Zinc finger nuclease (ZFN) recognizes its target sites which is composed of two zinc finger monomers that flank a short spacer sequence recognized by the FokI cleavage domain. **c** Transcription activator-like effector nuclease (TALEN) consists of two monomers; TALEN recognizes target sites which flank a fok1 nuclease domain to cut the DNA. **d** CRISPR/Cas9 system is made of a Cas9 protein with two nuclease domains: human umbilical vein endothelium cells (HuvC) split nuclease and the HNH, an endonuclease domain named for the characteristic histidine and asparagine residue, as well as a single guide RNA (sgRNA). (Adapted from [1, 51]; Gaj et al., 2016 [53];)

In the field of targeted nucleases and their potential application to model and non-model organisms, there are four major mechanisms of site-specific genome editing that have paved the way for new medical and agricultural breakthroughs. In particular, meganucleases (MegNs), zinc finger nucleases (ZFNs), transcription activator-like effector nuclease (TALENs), and clustered regularly interspaced short palindromic repeats (CRISPR)/CRISPR-associated protein 9 (Cas9) (CRISPR/Cas-9) (Fig. 2).

### Meganucleases (MegNs)

Meganucleases (MegNs) are naturally occurring endodeoxyribonucleases found within all forms of microbial life as well as in eukaryotic mitochondria and chloroplasts. The genes that encode MegNs are often embedded within self-splicing elements. The combination of molecular functions is mutually advantageous: the endonuclease activity allows surrounding introns and inteins to act as invasive DNA elements, while the splicing activity allows the endonuclease gene to invade a coding sequence without disrupting its product. The high specificity of these enzymes is based on their ability to cleave dsDNA at specific recognition sites comprising 14–40 bp (Fig. 2a). Unlike restriction enzymes, which provide defenses to bacteria against invading DNA, MegNs facilitate lateral mobility of genetic elements within an organism. This process is referred to as “homing” and gives the name homing endonucleases to these enzymes. The high DNA specificity of MegNs makes them a powerful protein scaffold to engineer enzymes for genome manipulation. A deep understanding of their molecular recognition of DNA is an important prerequisite to generate engineered enzymes able to cleave DNA in specific desired genome sites. Crystallographic analyses of representatives from all known MegNs families have illustrated both their mechanisms of action and their evolutionary relationships to a wide range of host proteins. The functional capabilities of these enzymes in DNA recognition vary widely across the families of MegNs. In each case, these capabilities, however, make a balance between what is called orthogonal requirements of (i) recognizing a target of adequate length to avoid overt toxicity in the host, while (ii) accommodating at least a small amount of sequence drift within that target. Indirect readout in protein-DNA recognition is the mechanism by which the protein achieves partial sequence specificity by detecting structural features on the DNA.

Several homing endonucleases have been used as templates to engineer tools that cleave DNA sequences other than their original wild-type targets.

Meganucleases can be divided into five families based on sequence and structure motifs: LAGLIDADG, GIY-YIG, HNH, His-Cys box, and PD-(D/E) XK [74]. I-CreI is a homodimeric member of MegNs family, which recognizes and cleaves a 22-bp pseudo-palindromic target (5′-CAAAACGTCGTGAGACAGTTTG-3′). The important role of indirect readout in the central region of the target DNA of these enzymes I-CreI suggested that indirect readout may play a key role in the redesign of protein-DNA interactions. The sequences of the I-CreI central substrate region, four bp (± 1 and ± 2) called 2NN, along with the adjacent box called 5NNN, are key for substrate cleavage [64]. Changes in 2NN significantly affect substrate binding and cleavage because this region affects the active site rearrangement, the proper protein-DNA complex binding, and the catalytic ion positioning to lead the cleavage.

An exhaustive review of each MegN can be found in Stoddard [75] as well as in Petersen and Niemann [63]. Several MegNs have been used as templates to engineer tools that cleave DNA sequences other than their original wild-type targets. This technology have advantages of high specificity of MegNs to target DNA because of their very long recognition sites, ease in delivery due to relatively small size, and giving rise to more recombinant DNA (i.e., more recombinogenic for HDR) due to production of a 3′ overhang after DNA cleavage. This lowers the potential cytotoxicity [53, 78].

Meganucleases have several promising applications; they are more specific than other genetic editing tools for the development of therapies for a wide range of inherited diseases resulting from nonsense codons or frameshift mutations. However, an obvious drawback to the use of natural MegNs lies in the need to first introduce a known cleavage site into the region of interest. Additionally, it is not easy to separate the two domains of MegNs: the DNA-binding and the DNA-cleavage domains, which present a challenge in its engineering. Another drawback of MegNs is that the design of sequence-specific enzymes for all possible sequences is time-consuming and expensive. Therefore, each new genome engineering target requires an initial protein engineering step to produce a custom MegN. Thus, in spite of the so many available MegNs, the probability of finding an enzyme that targets a desired locus is very small and the production of customized MegNs remains really complex and highly inefficient. Therefore, routine applications of MegNs in genome editing is limited and proved technically challenging to work with [24].

### Zinc finger nucleases (ZFNs)

The origin of genome editing technology began with the introduction of zinc finger nucleases (ZFNs). Zinc finger nucleases are artificially engineered restriction enzymes for custom site-specific genome editing. Zinc fingers themselves are transcription factors, where each finger recognizes 3–4 bases. Zinc finger nucleases are hybrid heterodimeric proteins, where each subunit contains several zinc finger domains and a Fok1 endonuclease domain to induce DSB formation. The first is zinc finger, which is one of the DNA binding motifs found in the DNA binding domain of many eukaryotic transcription factors responsible for DNA identification. The second domain is a nuclease (often from the bacterial restriction enzyme FokI) [6]. When the DNA-binding and the DNA-cleaving domains are fused together, a highly specific pair of “genomic scissors” is created (Fig. 2b). In principle, any gene in any organism can be targeted with a properly designed pair of ZFNs. Zinc finger recognition depends only on a match to DNA sequence, and mechanisms of DNA repair, both HR and NHEJ, are shared by essentially all species. Several studies have reported that ZFNs with a higher number of zinc fingers (4, 5, and 6 finger pairs) have increased the specificity and efficiency and improved targeting such as using modular assembly of pre-characterized ZFs utilizing standard recombinant DNA technology.

Since they were first reported [41], ZFN was appealing and showed considerable promise and they were used in several living organisms or cultured cells [11]. The discovery of ZFNs overcame some of the problems associated with MegNs applications. They facilitated targeted editing of the gene by inducing DSBs in DNA at specific sites. One major advantage of ZFNs is that they are easy to design, using combinatorial assembly of preexisting zinc fingers with known recognition patterns. This approach, however, suffered from drawbacks for routine applications. One of the major disadvantages of the ZFN is what is called “context-dependent specificity” (how well they cleave target sequence). Therefore, these specificities can depend on the context in the adjacent zinc fingers and DNA. In other terms, their specificity does not only depend on the target sequence itself, but also on adjacent sequences in the genome. This issue may cause genome fragmentation and instability when many non-specific cleavages occur. It only targets a single site at a time and as stated above. Although the low number of loci does not usually make a problem for knocking-out editing, it poses limitation for knocking in manipulation [32]. In addition, ZFNs cause overt toxicity to cells because of the off-target cleavages. The off-target effect is the probability of inaccurate cut of target DNA due to single nucleotide substitutions or inappropriate interaction between domains.

### Transcription activator-like effector nucleases (TALENs)

The limitations mentioned in the previous section paved the way for the development of a new series of nucleases: transcription activator-like effector nucleases (TALENs), which were cheaper, safer, more efficient, and capable of targeting a specified region in the genome [13].

In principle, the TALENs are similar to ZFNs and MegNs in that the proteins must be re-engineered for each targeted DNA sequence. The ZFNs and TALENs are both modular and have natural DNA-binding specificities. The TALEN is similar to ZFN in that it is an artificial chimeric protein that result from fusing a non-specific FokI restriction endonuclease domain to a DNA-binding domain recognizing an arbitrary base sequence (Fig. 2c). This DNA-binding domain consists of highly conserved repeats derived from transcription activator-like effectors (TALE). When genome editing is planned, a pair of TALEN is used like ZFNs. The TALE protein made of three domains: an amino-terminal domain having a transport signal, a DNA-binding domain which is made of repeating sequences of 34 amino acids arranged in tandem, and a carboxyl-terminal domain having a nuclear localization signal and a transcription activation domain. Of the 34 amino acids, there is a variable region of two amino acid residues located at positions 12 and 13 called repeat variable di-residues (RVD). This region has the ability to confer specificity to one of the any four nucleotide bps [15].

Unlike ZFNs, TALENs had advantages in that one module recognizes just one nucleotide in its DNA-binding domain, as compared with 3 bps recognized by the first single zinc finger domains [39]. So, interference of the recognition sequence does not occur even when several modules are joined. In theory, because cleavage of the target sequence is more specific than ZFN, it became possible to target any DNA sequence of any organism genome. This difference facilitates creation of TALEN systems which recognize more target sequences. Another benefit of the TALEN system over ZFN’s for genome editing is that the system is more efficient in producing DSBs in both somatic cells and pluripotent stem cells [35]. In addition, TALENs exhibit less toxicity in human cell lines due to off-target breaks that result in unwanted changes and toxicity in the genome. Another advantage of TALENs is a higher percentage of success in genome editing through cytoplasmic injection of TALEN mRNA in livestock embryos than observed with ZFN induction [39]. In addition, TALENs have been more successfully used in plant genome engineering [88]. It is hoped that TALENs will be applied in the generation of genetically modified laboratory animals, which may be utilized as a model for human disease research [24, 39].

The TALEN-like directed development of DNA binding proteins was employed to improve TALEN specificity by phage-assisted continuous evolution (PACE). The improved version was used to create genetically modified organisms [34]. Nucleases which contain designable DNA-binding sequences can modify the genomes and have the promise for therapeutic applications. DNA-binding PACE is a general strategy for the laboratory evolution of DNA-binding activity and specificity. This system can be used to generate TALEN with highly improved DNA cutting specificity, establishing DB-PACE as a diverse approach for improving the accuracy of genome editing tools. Thus, similar to ZFN, TALEN is used for DSBs as well as for knocking in/knocking out. In comparison with the ZFN, two important advantages for this editing technique have been reported: first, the simple design, and second, the low number of off-target breaks [35].

In spite of the improvement and simplification of the TALEN method, it is complicated for whom not familiar with molecular biological experiments. Moreover, it is confronted with some limitations, such as their large size (impeding delivery) in comparison to ZFN [24, 39]. The superiority of TALEN relative to ZFN could be attributed to the fact that in the TALEN each domain recognizes only one nucleotide, instead of recognizing DNA triplets in the case of ZEF. The design of TALEN is commonly more obvious than ZNF. This results in less intricate interactions between the TALEN-derived DNA-binding domains and their target nucleotides than those among ZNF and their target trinucleotides [35, 39].

### Clustered regularly interspaced short palindromic repeats (CRISPR)/CRISPR-associated protein 9 (Cas9)

The CRISPR/Cas system is the most recent platform in the field of genome editing. The system was developed in 2013 and is known as the third generation genomic editing tools. The clustered regularly interspaced short palindromic repeats, which are sometimes named “short regularly spaced repeats” were discovered in the 1980s. Computational analysis of these elements showed they were found in more than 40% of sequenced bacteria and 90% of archaea [37, 56]. The acronym CRISPR was suggested, and a group of genes adjacent to the CRISPR locus, which was termed “CRISPR-associated system”, or Cas was established [37]. Cas proteins coded by these genes carry functional domains similar to endonucleases, helicases, polymerases, and nucleotide-binding proteins. In addition, the role of CRISPRs as bacterial and archaeal adaptive immunity system against invading bacteriophages and other and in DNA repair was realized [17, 77].

Unlike the two previous technologies (ZFN and TALEN), in which the recognition of the DNA site was based on the sequence recognition by artificial proteins requiring interaction between protein and DNA, the DNA recognition of the CRISPR/Cas system is based on RNA-DNA interactions. This offers several advantages over ZFNs and TALENs. These include easy design for any genomic targets, easy prediction regarding off-target sites, and the probability of modifying several genomic sites simultaneously (multiplexing). CRISPR-Cas systems are diverse and have been classified thus far into two classes, six types, and over 20 subtypes based on locus arrangement and signature *cas* genes [33, 44, 51]. Types I, III, and IV, with multiprotein crRNA-effector complexes, are class 1 systems; types II, V, and VI, with a single protein-crRNA effector complex, are class 2. All CRISPR-Cas systems require Cas proteins and crRNAs for function, and CRISPR-*cas* expression is a prerequisite to acquire new spacers, process pre-crRNA, and assemble ribonucleoprotein crRNA interference complexes for target degradation. Herein, we will focus on the CRISPR-Cas9 technology, the reader should keep in mind other available variants of the system such as CRISPR-Cas6 [5], CRISPR-Cas12a, -Cas12b [42], as well as the most recently discovered c2c2 (Cas13a) and c2c6 (Cas13b [19, 69]. The CRISPR/Cas9 system is made of Cas9 nuclease and single-guide RNA (sgRNA). The sgRNA is an engineered single RNA molecule containing crispr RNA and tracr RNA parts. The sgRNA recognizes the target sequence by standard Watson-Crick base pairing. It has to be followed by a DNA motif called a protospacer adjacent motif (PAM). The commonly used wild-type *Streptococcus pyogenes* Cas (SpCas9) protein has a specific PAM sequence, 5’-NGG-3’, where “N” can be any nucleotide base followed by two guanine (“G”) nucleobases. This sequence is located directly downstream of the target sequence in the genomic DNA, on the non-target strand. Targeting is constrained to every 14 bp (12 bp from the seed sequence and 2 bp from PAM) [15]. SpCas9 variants may increase the specificity of genome modifications at DNA targets adjacent to NGG PAM sequences when used in place of wild-type SpCas9.

DNA cleavage is performed by Cas9 nuclease and can result in DSB in the the case of a wild-type enzyme, or in a SSB when using mutant Cas9 variants called nickases (Fig. 2d). It should be emphasized that the utilization of this approach in editing eukaryotes’ genome only needs the manipulation of a short sequence of RNA, and there is no need for complicated manipulations in the protein domain. This enables a faster and more cost-effective design of the DNA recognition moiety compared with ZFN and TALEN technologies. Applications of CRISPR-Cas9 systems are variable like those for ZFNs, TALENs, and MegNs. But, because of the relative simplicity of this system, its great efficiency and high tendency for multiple functions and library construction, it can be applied to different species and cell types [35].

**Fig. 3:**
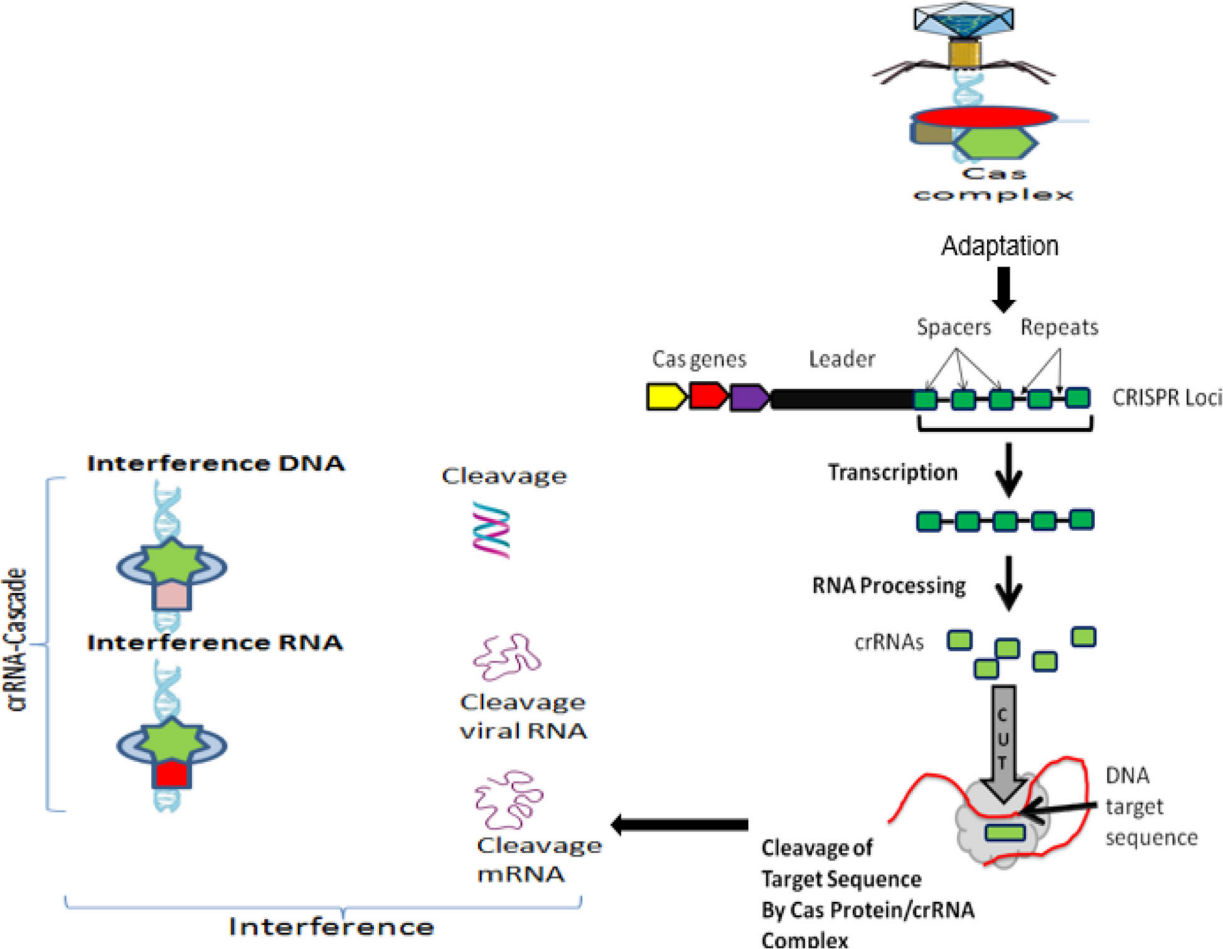
Schematic representation of CRISPR loci and targeting of DNA sequence, which include Cas genes, a leader sequence, and several spacer sequences derived from engineered or foreign DNA that are separated by short direct repeat sequences. The three major steps of CRISPR-Cas immune systems. In the adaptation phase, Cas proteins excise specific fragments from foreign DNA and integrate it into the repeat sequence neighboring the leader at the CRISPR locus. Then, CRISPR arrays are transcribed and processed into multiple crRNAs, each carrying a single spacer sequence and part of the adjoining repeat sequence. Finally, at the interference phase, the crRNAs are assembled into different classes of protein targeting complexes (cascades) that anneal to, and cleave, spacer matching sequences on either invading element or their transcripts and thus destroy them. (Adapted from [3, 53, 78])

As shown in Fig. 3, in all CRISPR/Cas systems, immunity occurs in three distinct stages [77, 81]: (1) adaptation or new spacer acquisition, (2) CRISPR transcription and processing (crRNA generation), and (3) interference or silencing. The advantages of the CRISPR/Cas system superseded those of both of the TALEN and ZFN tools, the ZFN in particular. This is due to its target design simplicity since the target specificity depends on ribonucleotide complex formation and non-protein/DNA recognition. In addition, the CRISPR/Cas approach is more efficient because changes can be introduced directly by injecting RNAs that encode the Cas protein and gRNA into developing embryos. Moreover, multigene mutations can be induced simultaneously by injecting them with multiple gRNAs. This is an example that explains the rapid spread of CRISPR/Cas 9 application in various fields. Still, the system has certain drawbacks. Although the CRISPR/Cas9 is much less complicated than TALEN, in terms of execution and construction, the off-target effect in CRISPR/Cas9 is higher than TALEN. Since the DSB results only after accurate binding of a pair of TALEN to the target sequence, the off-target effect problem is considered to be low. These two are different in restriction of target sequence. CRISPR/Cas9 is much more efficient than TALEN in multiple simultaneous modification. Table 1 compares the three main systems of site-directed synthetic nuclease employed in genome editing: ZFN, TALEN, and CRISPR/Cas9.

**Table 1:** Comparison of the three main currently used genome engineering platforms: ZFN, TALEN, and CRISPR/Cas9*

Aspect of comparison	ZFN	TALEN	CRISPR/Cas9
Origin	Eukaryotes	Bacteria	Bacteria/archaea
Structure	Dimer	Dimer	Monomer
Design simplicity	Moderate (ZFNs need customized protein for every DNA sequence)	Slightly complex (identical repeats are multiple, which creates technical issues of engineering and delivery into cells)	Simpler (available versions for crRNA can be easily designed)
Engineering feasibility/affordability	Low/limited	Moderate/affordable but resource intensive	High
Popularity/affordability	Low/limited	Moderate/affordable but resource intensive	High/highly affordable
DNA-binding molecule/DNA recognition mechanism	Zinc finger protein/protein-DNA interactions that introduce DSB	Transcription activator-like effectors/protein-DNA interactions that introduce DSB	crRNA or sgRNA/RNA-guided protein-DNA interactions that introduce DSB
Modification pattern	FokI nuclease	FokI nuclease	Cas9 nuclease
Specificity-determining length of recognition site	Typically 9–18 bp per ZFN monomer, 18–36 bp per ZFN pair	Typically 14–20 bp per TALEN monomer, 28–40 bp per TALEN pair	22 bp (20-bp guide sequence C 2-bp protospacer adjacent motif (PAM) for Cas9; up to 44 bp for double nicking
Targeting/target specificity	Low/difficult to target non-G-rich sequences/high; G-rich sequence preference; only small positional mismatches are tolerated; re-targeting requires protein engineering	Higher/for each TALEN monomer targeted base sequence must start (5′) with a T and end with an A (3’) end. High, requires a T at each 5’-end of its target; small positional mismatches are tolerated; re-targeting requires complex molecular cloning	Highest/targeted sequence end with an NGG or NAG (lower activity) sequence (that is, PAM) Moderate: RNA-targeted sequence must precede the 2 base pairs recognized by PAM. Only small positional and multiple consecutive mismatches are tolerated. Re-targeting requires new RNA guide. Protein engineering is not required.
Mechanism of action	Introduction of double-strand breaks (DSBs) in target DNA	Introduction of double-strand breaks (DSBs) in target DNA	Introduction of DSBs in target DNA by wtCas9 or single-strand nicks by Cas9 nickase
Cleavage efficacy	Efficient	Efficient	Highly efficient
Multiplex genome editing	Not easy (few models)	Not easy (few models)	Easy (high-yield multiplexing available (no need for obtaining embryonic stem cells))
Delivery vehicle	Easy via electroporation and viral vectors transduction	Easy in vitro delivery; difficult in vivo due to the large size of TALEN DNA and their high probability of recombination	Easy in vitro; moderate difficulty of delivery in vivo due to poor packaging of the large Cas9 by viral vectors.
Use as gene activator	Yes; activation of endogenous genes; minimal off-target effects; may require engineering to target particular sequences	Yes; activation of endogenous genes; minimal off-target effects; no sequence limitations	Yes; activation of endogenous genes; minimal off-target effects; requires “NGG” PAM next to the target sequence
Use as gene inhibitor	Yes; works by blocking transcription elongation via chromatin repression; minimal off-target effects; may require engineering to target particular sequences	Yes; works by blocking transcription elongation via chromatin repression; minimal off-target effects; no sequence limitations	Yes; works by blocking transcription elongation via chromatin repression; minimal off-target effects; requires “NGG” PAM next to target sequence.
Success rate‡	Low (~ 24%)	High (> 99%)	High (~ 90%)
Average mutation rate§	Low or variable (~ 10%)	High (~ 20%)	High (~ 20%)
Off-target effects	Highly possible off-target activities	Low possible off-target activities	Variable; limited off-target activities, not fully studied in plants
Programmable	Highly difficult	Difficult	Easy
Cytotoxicity	Variable to high	Low	Low
Cost	Low	High	Low
Online resources for nuclease design	• The Zinc Finger Consortium includes software tools and protocols genome-wide tag scanner for nuclease off-sites • The Segal Laboratory software site • ZFN target site algorithm for identifying sites compatible with the Lawson-Wolfe modular assembly system • Zinc finger tools • ZiFiT Targeter software	• E-TALEN • Genome engineering resources • Scoring algorithm for predicting TALE(N) activity • ToolGen TALEN designer • ZiFiT Targeter software	• E-CRISP • Genome engineering resources • RGEN tools • ZiFiT Targeter software
Suppliers Non-profit organizations *Companies	- Addgene (*https://www.addgene.org/*) *Sigma-Aldrich/ToolGen	- Addgene/TALEN library resource *Cellectis Bioresearch/Life Technologies/ToolGen/Transposagen Biopharmaceuticals	- Addgene *Life Technologies/Sigma-Aldrich/System Biosciences/ToolGen/Transposagen Biopharmaceuticals

A wide range of success rates and mutation rates (which depend on factors such as the methods used to construct these nucleases, delivery methods, and cell lines or organisms) have been reported. Mutation frequencies are higher in K562 cells and HeLa cells than in HEK293 cells

*Abbreviations: *Cas9* CRISPR (clustered regularly interspaced short palindromic repeat)-2 associated protein 9, *crRNA* CRISPR RNA, *N* any nucleotide, *PAM* protospacer adjacent motif, *RGEN* RNA-guided engineered nuclease, *sgRNA* single-chain guide RNA, *TALEN* transcription activator-like effector nuclease, *ZFN* zinc finger nuclease

‡The success rate is defined as the proportion of nucleases that induce mutations at frequencies > 0.5% in HEK293 cells

§The average mutation rate is based on the frequency of non-homologous end-joining-mediated insertions and deletions obtained at the nuclease target site [1, 39, 48, 78]. The Innovative Genomics Institute (https://innovativegenomics.org/) is another excellent source of background information, explainers, and a terrific glossary with fun animations (https://innovativegenomics.org/resources/educational-materials/)

The off-target effect is an essential subject for future studies if CRISPR/Cas9 is to achieve its promises as a powerful method for genome editing. Non-specific and unintended genetic modifications (off-target effect) can result from the use of CRISPR/Cas9 system which is one of the drawbacks of this tool. Therefore, this point should be considered for use in researches. One strategy to reduce the off-target activity is to replace the *Streptococcus pyogenes* Cas9 enzyme (SpyCas9) for a mutant Cas9 nickase (nSpyCas9; ncas9), which cleaves a single strand through the inactivation of a nuclease domain Ruvc or HNH [9]. Our understanding of off-target effects remains fragmentary. A deeper understanding of this phenomenon is needed. Several approaches that could be followed to characterize the binding domains and consequently Cas9 targeting specificity have been reviewed and summarized [83].

**Fig. 4:**
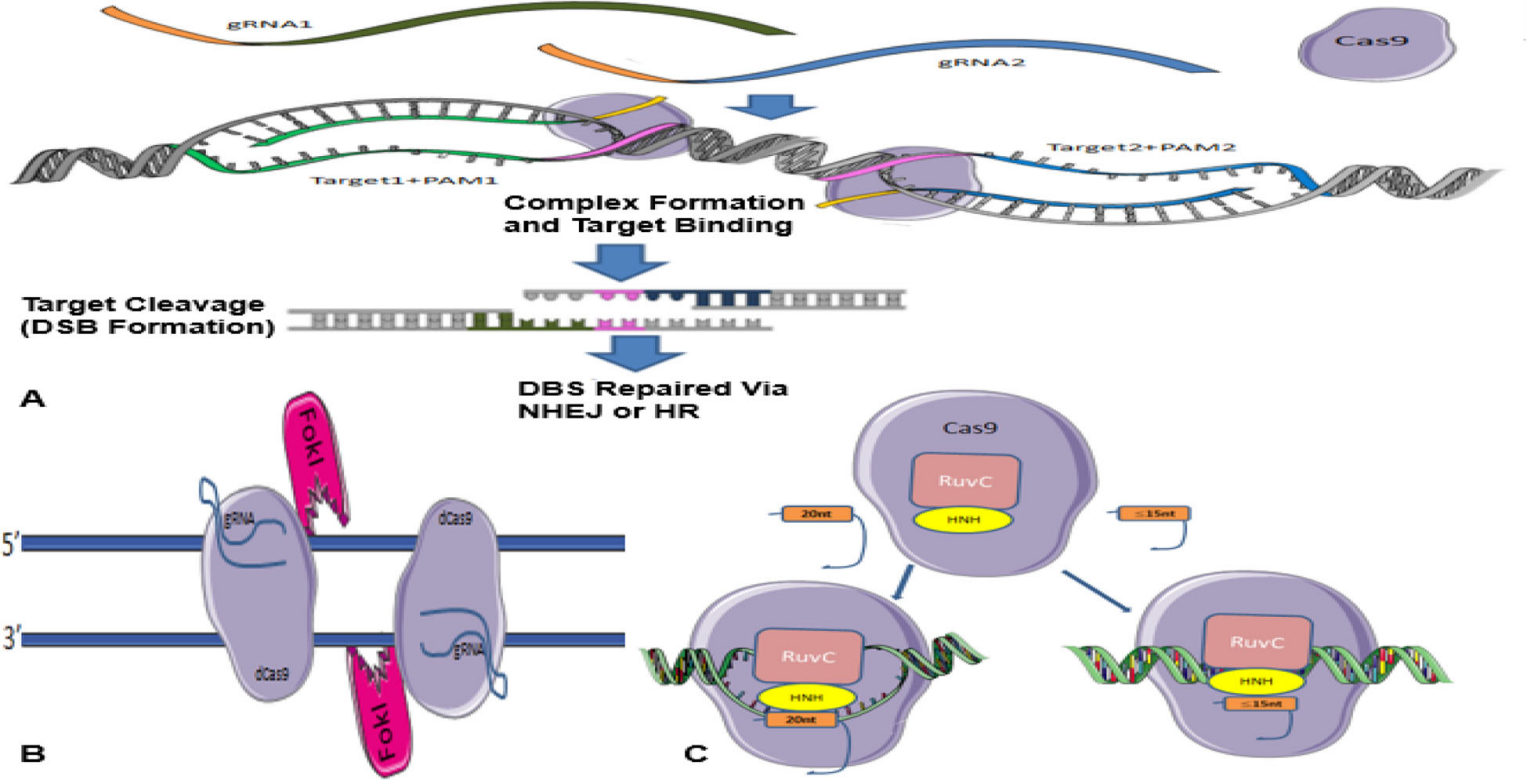
**a** Summary of the Cas9 nickases methods in efficient genome editing. Two gRNAs target opposite strands of DNA. These double nicks create a DSB that is repaired using non-homologous end joining (NHEJ) or edits via homology-directed repair (HDR) (adapted from www.addgene.org/crispr/nick). **b** FokI-dCas 9 fusion protein method. Two FokI-dCas9 fusion proteins are used to adjacent target sites by two different sgRNAs to facilitate FokI dimerization and DNA cleavage. These fusions would have enhanced specificity compared to the standard monomeric Cas9 nucleases and the paired nickase system because they should require two sgRNAs for activity. **c** Truncated sgRNA method. Cas9 interacting with either a full-length sgRNA (20 nucleotide sequence complementary to target site) or truncated gRNA (less than 15 nucleotide sequence complementary to target site). (Retrieved from blog.addgene.org)

It has previously been stated that CRISPR/Cas9 system needs both gRNA and PAM to detect its target sequence of interest by integration of a gRNA component that binds to complementary double-stranded DNA sequences. Cell culture studies have shown that off-target effects may be due to the incorrect detection of genomic sequences by sgRNA. This, in turn, affects cleavage when the mismatch is in the vicinity of the PAM (up to 8 bases), but if the PAM is too far apart, these effects will be small [4], even a slight mismatch between sgRNA and target sequences can lead to a failure. Dependence of this method on specific PAM sequences to act functionally limits the number of target loci, and it can reduce off-target breaks [86]. For this goal, another type of specific PAM-containing nucleases has been prepared to compensate for this limitation. Genetic engineering and enzyme changing have also been able to overcome the limitation [42]. For a sgRNA, many similar sequences depending on the genome size of the species may exist [86]. Interestingly, the initial targeting scrutiny of the CRISPR/Cas9-sgRNA complex showed that not every nucleotide base in the gRNA is necessary to be complementary to the target DNA sequence to effect Cas9 nuclease activity. Regarding that where the similar sequences are found in the genome, their breaks could lead to malignancies or even death [86]. Various methods have been proposed to prevent off-target breaks, among which the double nicking method, the FokI-dCas9 fusion protein method, and the truncated sgRNA method [76] (Fig. 4).

**Fig. 5:**
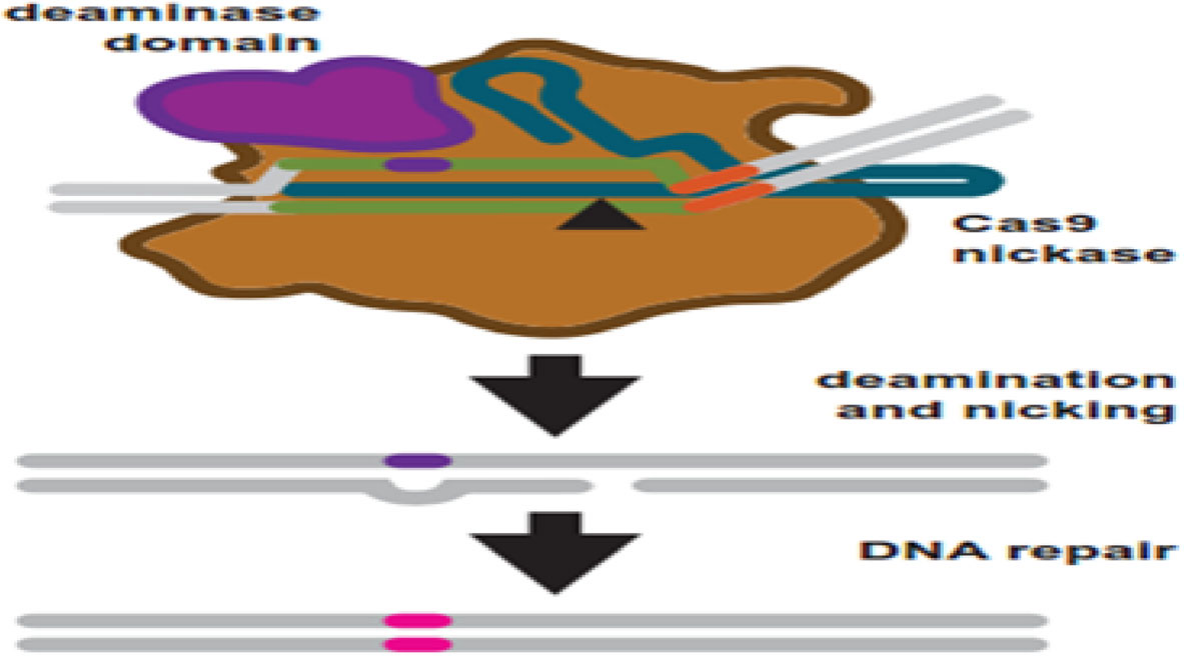
Base editing uses engineered Cas9 variants to induce base changes in a target sequence. Cas9 nickase is fused to a base deaminase domain. The deaminase domain works on a targeted region within the R-loop after target binding and R-loop formation. Simultaneously, the target strand is nicked. DNA repair is started in response to the nick using the strand which contains the deaminated base as a repair template. Repair leads to a transition mutations: C:G to T:A and A:T to G:C for cytosine and adenosine base editors, respectively [68]

**Fig. 6:**
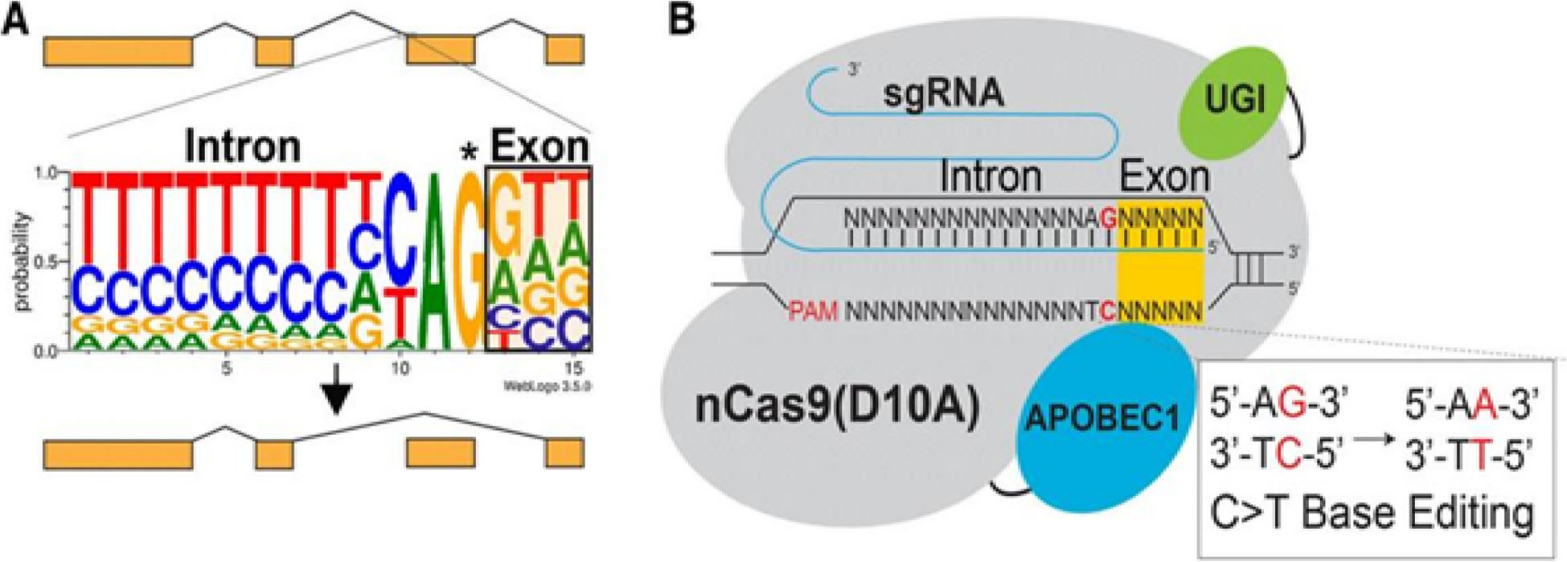
Essential steps in CRISPR-SKIP targeting approach: **a** Nearly every intron ends with a guanosine (asterisked G). It is hypothesized that mutations that disrupt this highly conserved G within the splice acceptor of any given exon in genomic DNA would lead to exon skipping by preventing incorporation of the exon into mature transcripts base. **b** In the presence of an appropriate PAM sequence, this G can be effectively mutated by converting the complementary cytidine to thymidine using CRISPR-Cas9 C>T single-base editors. (From [25])

To overcome these problems, researchers explored another generation of base editing technologies, which combine CRISPR and cytidine deaminase (Fig. 5). This is a diverse method called CRISPR-SKIP (Fig. 6) which uses cytidine deaminase single-base editors to program exon skipping by mutating target DNA bases within splice acceptor sites [25]. Given its simplicity and precision, CRISPR-SKIP will be widely applicable in gene therapy. Base editing utilizes Cas9 D10A nickases fused to engineered base deaminase enzymes to make single base changes in the DNA sequence without the need of DNA DSB. Also, base editing does not require an external repair template. The Cas9 nickase part of the base editor protein plays a dual function. The first is to target the deaminase activity to the wanted region and the second is to localize the enzyme to certain regions of double-stranded RNA. The deaminase domains in base editors (BEs) occur in two versions: either adenosine deaminase or cytosine deaminase, which catalyze only base transitions (C to T and A to G) and cannot produce base transversions [26, 68]. In these base editing tools, the targeted activity of adenosine deaminase can result in an A:T to G:C sequence alteration in a very similar way [26, 68].This approach avoided the requirement of breaking DNA to induce an oligonucleotide. In addition, compared to knocking system, it exerted a higher output with lower off-targets [40, 43]. Adenosine is deaminated to inosine (I) that is subsequently utilized to repair the nicked strand with a cytosine, and the I:C base pair is resolved to G:C [26]. More recently, new genome editing technologies have been developed: glycosylase base editors (GBEs), which consist of a Cas9 nickase, a cytidine deaminase, and a uracil-DNA glycosylase (Ung), are capable of transversion mutations by changing C to A in bacterial cells and from C to G in mammalian cells [45, 89]. The new BEs can also be designed to minimize unwanted (“off-target”) mutations that could potentially cause undesirable side effects. The novel BE platform may help researchers understand and correct genetic diseases by selective editing of single DNA “alphabets” across nucleobase classes. However, the technique with this new class of transversion BEs is still at an early stage and requires additional optimization, so it would be premature to say this is ready for the clinic applications.

## Gene delivery

From biotechnology’s point of view, the main obstacle that is facing molecular technology is to select the right method that is simple but effective to transfer the gene to the host cell. The components of gene editing have to be transferred to the cell/nucleus of interest using in vivo, ex vivo, or in vitro route. In this regard, several concerns must be considered including physical barriers (cell membranes, nuclear membranes) as well as digestion by proteases or nucleases of the host. Another important issue is the possible rejection by the immune system of the host if the components are delivered in vivo. In general, the gene delivery routes can be categorized in three classes of physical delivery, viral vectors, and non-viral agents. Although the direct delivery of construct plasmids may sound easy and more efficient and specific than the physical and the chemical methods, it proves to be an inappropriate choice because the successful gene delivery system requires the foreign genetic molecule to remain stable within the host cells [52]. The other possible procedure is to use viruses. However, because plant cells have thick walls, the gene transfer systems for plants involve transient and stable transformation using protoplast-plasmid in vitro [54]: agrobacterium-mediated transformation, gene gun and viral vectors (transient expression by protoplast transformation), and agro-infiltration [1]. Viruses may present a suitable vehicle to transfer genome engineering components to all plant parts because they do not require transformation and/or tissue culture for delivering and mutated seeds could easily recovered. For many years, scientists employed different species of *Agrobacterium* to systematically infect a large number of plant species and generate transgenic plants. These bacterial species have small genome size and this facilitates cloning and agroinfections, and the virus genome does not integrate into plant genomes [1].

Of the challenges and approaches of delivering CRISPR, it was pointed out [18, 51] that although the present genome engineering is in favor of CRISPR tools, TALENs may still be of a primary choice in certain experimental species. For example, TALENs have been utilized in targeted genomic editing in *Xenopus tropicalis* by knocking-out Klf4 [49, 50] or thyroid hormone receptor α [23]. In addition, TALENs have been utilized to modify genome of human stem cells [47]. Also TALEN approach has been applied to create amniotic mesenchymal stem cells overexpressing anti-fibrotic interleukin-10 [12]. Lately, a geminivirus genome has been prepared to deliver various nucleases platforms (including ZFN, TALENs, and the CRISPR/Cas system) and repair template for HR of DSBs [62].

To deliver the carrying DNA sequence to target cells, non-viral techniques such as electroporation, lipofection, and microinjection can also be used [18]. In addition, these techniques also reduce off-target cleavages problems. Gene transfer via microinjection is considered the gold standard procedure since its efficiency is approximately 100% [85]. The advantage of this approach is its high efficacy and less constrains on the size of the delivery. A disadvantage is that it can be employed only in in vitro or ex vivo cargo. Recently, small RNAs, including small interfering RNA (siRNA) and microRNA (miRNA), have been widely adopted in research to replace laboratory animals and cell lines. Development of innovative nanoparticle-based transfer systems that deliver CRISPR/Cas9 constructs and maximize their effectiveness has been tested in the last few years [29, 58].

## Applications of gene technology

The ability of the abovementioned gene delivery systems to target and manipulate the genome of living organisms has been attractive to many researchers worldwide. Despite all limitations, the interest in this technology has developed its capabilities and enhanced its scope of applications. Genome/gene engineering technology is relatively applicable and has potential to effectively and rapidly revolutionize genome surgery and will soon transform agriculture, nutrition, and medicine. Some of the most important applications are briefly described below.

### Plant-based genome editing

The appearance of genome editing has been appealing especially to agricultural experts. One of the major goals for utilizing genome editing tools in plants is to generate improved crop varieties with higher yields and clear-cut addition of valuable traits such as high nutritional value, extended shelf life, stress tolerance, disease and pest resistance, or removal of undesirable traits [1]. However, several obstacles related to the precision of the genetic manipulations and the incompatibility of the host species have hampered the development of crop improvements [2]. The use of site-specific nucleases is one of the important promising techniques of gene editing that helped overcome certain limitations by specifically targeting a suitable site in a gene/genome. The employment of the gene editing technologies, including those discussed in this review, seems to be endless ever since their emergence, and several improvements in original tools have further brought accuracy and precision in these methods [78].

### Animal-based genome editing

Recent genome editing techniques has been extensively applied in many organisms, such as bacteria, yeast, and mouse [53, 73]. Genetic manipulation tools cover a wide range of fields, including the generation of transgenic animals using embryonic stem cells (ESC), functional analysis of genes, model development for diseases, or drug development. Genome editing techniques have been used in many various organisms. Among the livestock and aquatic species, ZFN is only used for zebrafish, but two other technologies, TALEN and CRISPR, have been used at the cell level in chicken, sheep, pig, and cattle. Engineered endonucleases or RNA-guided endonucleases (RGENs) mediated gene targeting has been applied directly in a great number of animal organisms including nematodes and zebrafish [20, 57], as well as pigs [71, 85]. Since the first permission to use CRISPR/Cas9 in human embryos and in vivo genome editing via homology-independent targeted integration (HITI), an increasing number of studies have identified striking differences between mouse and human pre-implantation development and pluripotency [66], highlighting the need for focused studies in human embryos. Therefore, more specific criteria and widely accepted standards for clinical research have to be met before human germline editing would be deemed permissible [31]. In this regard, results of some research on the *human genome editing* have been questioned. The “He Jiankui experiments at the beginning of 2019”, which claimed to have created the world’s first genetically edited babies, is simply the most recent example. He Jiankui said he edited the babies’ genes at conception by selecting CRISPR/cas9 to edit the chemokine receptor type 5 (CCR5) gene in cd4+ cells in hopes of making children resistant to the AIDS virus, as their father was HIV-positive. Researchers said He’s actions exposed the twins to unknown health risks, possibly including a higher susceptibility to viral illnesses. For more information on the scientific reactions around the world, the reader may find helpful several excellent sources of information [38, 49, 79, 84].

### Gene therapy

The original principles of gene therapy arose during the 1960s and early 1970s when restriction enzymes were utilized to manipulate DNA [22]. Since then, researchers have done great efforts to treat genetic diseases but treatment for multiple mutations is difficult. Different clinical therapy applications have been attempted to overcome these problems. Much of the interest in CRISPR and other gene editing methods revolves around their potential to cure human diseases. It is hoped that eradication of human diseases is not too far to achieve via the CRISPR system because it was employed in other fields of biological sciences such as genetic improvement and gene therapy. It is important to mention that the therapeutic efficiency of gene editing depends on several factors, such as editing efficacy, which varies widely depending on the cell type, senescence status, and cell cycle status of the target [69]. Other factors that also influence therapeutic effectiveness include cell aptitude, which refers to the feasibility of accomplishing a therapeutic modification threshold, and the efficient transfer of programmable nuclease system to the target tissue, which is only considered to be effective if the engineered nuclease system reaches safely and efficiently to the nucleus of the target cell. Finally, the precision of the editing procedure is another important aspect, which refers to only editing the target DNA without affecting any other genes [80].

**Fig. 7:**
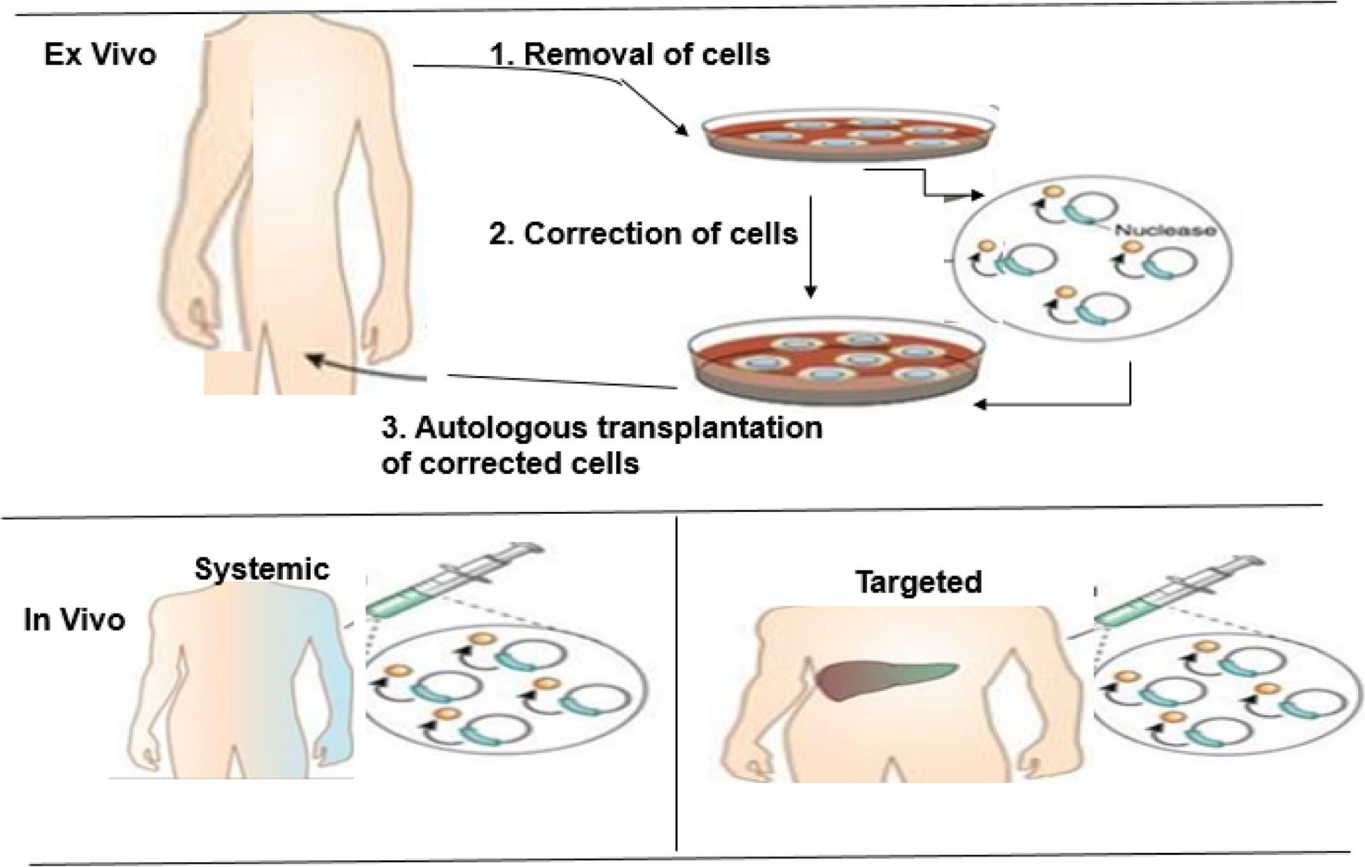
Outline of the ex vivo and in vivo genome editing procedures for clinical therapy. Top: In the ex vivo editing therapy, cells are removed from a patient to be treated, corrected by gene editing and then re-engrafted back to the patient. To achieve therapeutic success, the target cells must be capable of surviving in vitro and autologous transplantation of the corrected cells. Below: In the in vivo editing therapy, designed nucleases are administered using viral or non-viral techniques and directly injected locally to the affected tissue, such as the eye, brain, or muscle. (Adapted from [48])

The genome editing tools have enabled scientists to utilize genetically programmed animals to understand the cause of various diseases and to understand molecular mechanisms that can be explored for better therapeutic strategies (Fig. 7). Genome editing gives the basis of the treatment of many kinds of diseases. In preliminary experiments, the knocking-in procedure was used to reach this goal. There are examples of gene editing techniques applied in different genetic diseases in cell lines, disease models, and human [48, 53, 82]. These encouraging results suggest the therapeutic capability of these gene editing strategies to treat human genetic diseases including Duchenne muscular dystrophy [8, 28, 55], cystic fibrosis [21], sickle cell anemia [62], and Down syndrome [7]. In addition, this technology has been employed in curing Fanconi anemia by correcting point mutation in patient-derived fibroblasts [60], as well as in hemophilia for the restoration of factor VIII deficiency in mice [61, 87]. The CRISPR tools have also demonstrated promising results in diagnosis and curing fatal diseases such as AIDS and cancer [16, 30, 84].

### Other applications

The applications mentioned above were more about knock out or modification of genes Gapinske et al. [25]. However due to inactivate nuclease activity nature of the dCas9, CRISPR can be used in other applications as well. By selecting the target sequence, gene expression can be controlled by inhibiting the transcription rate of RNA polymerase II (polII) or inhibiting the transcription factor binding [65]. Additionally, combining gene expression inhibitors such as Krüppel-associated box with the inactivated Cas9 has led to generate a special kind of gene inhibitors, which are called CRISPR interference (CRISPRi), and downregulate gene expression [46]. It is also possible to control gene expression by fusing transcription-activating molecule, the transcription-repressing molecule, or the genome-modifying molecule to dCas9 [27].

## Conclusions

Genome editing is a fast-growing field. Editing nucleases have revolutionized genomic engineering, allowing easy editing of the mammalian genome. Much progress has been accomplished in the improvement of gene editing technologies since their discovery. Of the four major nucleases used to cut and edit the genome, each has its own advantages and disadvantages, and the choice of which gene editing method depends on the specific situation. The current genome editing techniques are still buckling up with problems, and it is difficult to perform genome editing in cells with low transfection efficiency or in some cultured cells such as primary cultured cells. Genotoxicity is an inherent problem of enzymes that act on nucleic acids, though one can expect that highly specific endonucleases would reduce or abolish this issue. Exceptional efforts are needed in future to complement and offer something novel approaches in addition to the already existing ones. It is anticipated that research in gene editing is going to continue and tremendously advance. With the development of next-generation sequencing technology, new extremely important clinical applications, such as manufacturing engineered medical products, eradication of human genetic diseases, treatment of AIDS and cancers, as well as improvement of crop and food, will be introduced. Combination of genomic modifications induced by targeted nucleases to their own self-degradation, self-inactivating vectors may help overcoming confronting limitations discussed above to improve the specificity of genome editing, especially because the frequency of off-target modifications. Our understanding of off-target effects remains poor. This is a vital area for continued study if CRISPR/Cas9 is to realize its promise. Regarding gene cargo delivery systems, this remains the greatest obstacle for CRISPR/Cas9 use, and an all-purpose delivery method has yet to emerge. The union between genome engineering and regenerative medicine is still in its infancy; realizing the full potential of these technologies in reprograming the fate of stem/progenitor cells requires that their functional landscape be fully explored in these genetic backgrounds. Humankind can only wait to see what the potential of these technologies will be. One major question is whether or not the body’s immune response will accept or reject the foreign genetic elements within the cells. Another important concern is that along with the revolutionary advances of this biotechnology and related sciences, bioethical concerns and legal problems related to this issue are still increasing in view of the possibility of human genetic manipulation and the unsafety of procedures involved [49, 50, 66]. The enforcement of technical and ethical guidelines, and legislations should be considered and need serious attention as soon as possible.

## Data Availability

Not applicable

## Abbreviations

Cas-9: CRISPR-associated protein 9
CRISPR: Clustered regularly interspaced short palindromic repeats
crRNA: CRISPR RNA
DSB: Double-stranded break
ESC: Embryonic stem cells
HDR: Homology-directed repair
HITI: Homology-independent targeted integration
HR: Homologous recombination
HuvC: Human umbilical vein endothelium cells
I-Sce I: Intron-encoded endonuclease
MegNs: Meganucleases
MMEJ: Microhomology-mediated end joining
NHEJ: Non-homologous end joining
PACE: Phage-assisted continuous evolution
PAMs: Protospacer adjacent motifs
RGENs: RNA-guided endonucleases
RVD: Repeat variable di-residues
sgRNA: Single guide RNA
SpyCas9: *Streptococcus pyogenes* Cas9
SSB: Single-strand break
TALENs: Transcription activator-like effector nuclease
ZFNs: Zinc finger nucleases

## References

[1] Abdallah N, Prakash C, Mchughen A (2015). Genome editing for crop improvement: challenges and opportunities. GM Crops Food.

[2] Aglawe S, Barbadikar K, Mangrauthia S, Madhav M (2018). New breeding technique “genome editing” for crop improvement: applications, potentials and challenges. 3 Biotech.

[3] Alkhnbashi OS, Fabrizio C, Shah SA, Garrett RA, Saunders SJ, Rolf B (2014). CRISPR strand: predicting repeat orientations to determine the crRNA-encoding strand at CRISPR loci. Bioinformatics.

[4] Anders C, Niewoehner O, Duerst A, Jinek M (2014). Structural basis of PAM-dependent target DNA recognition by the Cas9 endonuclease. Nature.

[5] Bernal-Bernal D, Abellón-Ruiz J, Iniesta AA, Pajares-Martínez E, Bastida-Martínez E, Fontes M (2018). Multifactorial control of the expression of a CRISPR-Cas system by an extracytoplasmic function σ/anti-σ pair and a global regulatory complex. Nucleic Acids Res.

[6] Bibikova M, Carroll D, Segal DJ, Trautman JK, Smith J, Kim YG (2001). Stimulation of homologous recombination through targeted cleavage by chimeric nucleases. Mol Cell Biol.

[7] Bloh KM, Bialk PA, Gopalakrishnapillai A, Kolb EA, Kmiec EB (2017). CRISPR/Cas9-directed reassignment of the GATA1 initiation codon in K562 cells to recapitulate AML in Down syndrome. Mol Ther Nucleic Acids.

[8] Cai A, Kong X (2019) Development of CRISPR-mediated systems in the study of Duchenne muscular dystrophy. Hum Gene Therap Methods 10.1089/hgtb.2018.187 10.1089/hgtb.2018.187 31062609

[9] Cao J, Wu L, Zhang SM, Lu M, Cheung WK, Cai W, Gale M (2016). An easy and efficient inducible CRISPR/Cas9 platform with improved specificity for multiple gene targeting. Nucleic Acids Res.

[10] Capecchi MR (1989). Altering the genome by homologous recombination. Science.

[11] Carroll D (2011). Genome engineering with zinc-finger nucleases. Genetics.

[12] Choi J, Jeong I, Han J, Cheon S, Kim S (2019). IL-10-secreting human MSCs generated by TALEN gene editing ameliorate liver fibrosis through enhanced anti-fibrotic activity. Biomater Sci.

[13] Christian ML, Demorest ZL, Starker CG, Osborn MJ, Nyquist MD, Zhang Y (2012). Targeting G with TAL effectors: a comparison of activities of TALENs constructed with NN and NK repeat variable di-residues. PLoS One.

[14] Cohen-Tannoudji M, Robine S, Choulika A, Pinto D, El Marjou F, Babinet C (1998). I-sceI-induced gene replacement at a natural locus in embryonic stem cells. Mol Cell Biol.

[15] Cong L, Ran FA, Cox D, Lin S, Barretto R, Habib N (2013). Multiplex genome engineering using CRISPR/Cas systems. Science.

[16] Cyranoski D (2016). CRISPR gene-editing tested in a person for the first time. Nature.

[17] Doudna JA, Charpentier E (2014). The new frontier of genome engineering with CRISPR-Cas9. Science.

[18] Dumeau C-E, Monfort A, Kissling L, Swarts DC, Jinek M, Wutz A (2019). Introducing gene deletions by mouse zygote electroporation of Cas12a/Cpf1. Transgenic Res.

[19] East-Seletsky A, O’Connell MR, Burstein D, Knott GJ, Doudna JA (2017). RNA targeting by functionally orthogonal Type VI-A CRISPR-Cas enzymes. Mol Cell.

[20] Fernandez JP, Vejnar CE, Giraldez AJ, Rouet R, Moreno-Mateos MA (2018). Optimized CRISPR-Cpf1 system for genome editing in zebrafish. Methods.

[21] Firth AL, Menon T, Parker GS, Qualls SJ, Lewis BM, Ke E (2015). Functional gene correction for cystic fibrosis in lung epithelial cells generated from patient iPSCs. Cell Rep.

[22] Friedmann T, Roblin R (1972). Gene therapy for human genetic disease?. Science.

[23] Fu L, Wen L, Shi Y (2018). Role of thyroid hormone receptor in amphibian development. Methods Mol Biol.

[24] Gaj T, Sirk SK, S-l S, Liu J (2016). Genome-editing technologies: principles and applications. Cold Spring Harb Perspect Biol.

[25] Gapinske M, Luu A, Winter J, Woods ES, Kostan KA, Shiva N (2018). CRISPR-SKIP: programmable gene splicing with single base editors. Genome Biol.

[26] Gaudelli NM, Komor AC, Rees HA, Packer MS, Badran AH, Bryson DI (2017). Programmable base editing of A•T to G•C in genomic DNA without DNA cleavage. Nature.

[27] Gilbert LA, Larson MH, Morsut L, Liu Z, Brar GA, Torres SE, Stern-Ginossar N, Brandman O, Whitehead EH, Doudna JA, Lim WA, Weissman JS, Qi LS (2013). CRISPR-mediated modular RNA-guided regulation of transcription in eukaryotes. Cell.

[28] Ginn SL, Amaya AK, Alexander IE, Edelstein M, Abedi MR (2018). Gene therapy clinical trials worldwide to 2017: an update. J Gene Med.

[29] Givens BE, Naguib YW, Geary SM, Devor EJ, Salem AK (2018). Nanoparticle-based delivery of CRISPR/Cas9 genome-editing therapeutics. AAPS J.

[30] Gootenberg JS, Abudayyeh OO, Kellner MJ, Joung J, Collins JJ, Zhang F (2018). Multiplexed and portable nucleic acid detection platform with Cas13, Cas12a, and Csm6. Science.

[31] Greely HT (2019). CRISPR’d babies: human germline genome editing in the ‘He Jiankui affair’. J Law Biosci.

[32] Gupta A, Hall VL, Kok FO, Shin M, McNulty JC, Lawson ND, Wolfe SA (2013). Targeted chromosomal deletions and inversions in zebrafish. Genome Res.

[33] Hille F, Richter H, Wong SP, Bratovic M, Ressel S, Charpentier E (2018). The biology of CRISPR-Cas: backward and forward. Cell.

[34] Hubbard BP, Badran AH, Zuris JA, Guilinger JP, Davis KM, Chen L (2015). Continuous directed evolution of DNA-binding proteins to improve TALEN specificity. Nat Methods.

[35] Huo Z, Tu J, Xu A, Li Y, Wang D, Liu M (2019). Generation of a heterozygous p53 R249S mutant human embryonic stem cell line by TALEN-mediated genome editing. Stem Cell Res.

[36] Ishino Y, Shinagawa H, Makino K, Amemura M, Nakata A (1987). Nucleotide sequence of the iap gene, responsible for alkaline phosphatase isozyme conversion in Escherichia coli, and identification of the gene product. J Bacteriol.

[37] Jansen R, Embden JD, Gaastra W, Schouls LM (2002). Identification of genes that are associated with DNA repeats in prokaryotes. Mol Microbiol.

[38] Jonlin EC (2020). Informed consent for human embryo genome editing. Stem Cell Rep.

[39] Khan SH (2019). Genome-editing technologies: concept, pros, and cons of various genome-editing techniques and bioethical concerns for clinical application. Mol Ther Nucleic Acids.

[40] Kim K, Bang SY, Lee HS, Bae SC (2017). Update on the genetic architecture of rheumatoid arthritis. Nat Rev Rheumatol.

[41] Kim Y-G, Cha J, Chandrasegaran S (1996). Hybrid restriction enzymes: zinc finger fusions to Fok I cleavage domain. Proc Natl Acad Sci U S A.

[42] Kleinstiver BP, Sousa AA, Walton RT, Tak YE, Hsu JY (2020). Engineered CRISPR-Cas12a variants with increased activities and improved targeting ranges for gene epigenetic and base editing. Nat Biotechnol.

[43] Komor AC, Zhao KT, Packer MS, Gaudelli NM, Waterbury AL, Koblan LW et al (2017) Improved base excision repair inhibition and bacteriophage Mu Gam protein yields C:G-to-T:A base editors with higher efficiency and product purity. Sci Adv 3:eaao4774 10.1126/sciadv.aao4774 PMC5576876 28875174

[44] Koonin EV, Makarova KS, Zhang F (2017). Diversity, classification and evolution of CRISPR-Cas systems. Curr Opin Microbiol.

[45] Kurt IC, Zhou R, Iyer S, Garcia SP, Miller BR, Langner LM, Grünewald J, Joung JK (2020) CRISPR C-to-G base editors for inducing targeted DNA transversions in human cells. Nat Biotechnol 10.1038/s41587-020-0609-x 10.1038/s41587-020-0609-x PMC7854778 32690971

[46] Larson MH, Gilbert LA, Wang X, Lim WA, Weissman JS, Qi LS (2013). CRISPR interference (CRISPRi) for sequence-specific control of gene expression. Nat Protoc.

[47] Lee J, Termglinchan V, Diecke S, Itzhaki I, Lam C, Garg P (2019). Activation of PDGF pathway links LMNA mutation to dilated cardiomyopathy. Nature.

[48] Li H, Yang Y, Hong W, Huang M, Wu M, Zhao X (2020) Applications of genome editing technology in the targeted therapy of human diseases: mechanisms, advances and prospects. Sig Transduct Target Ther 5(1) 10.1038/s41392-019-0089-y 10.1038/s41392-019-0089-y PMC6946647 32296011

[49] Li J-R, Walker S, Nie J-B, Xin-qing Zhang X-Q (2019). Experiments that led to the first gene-edited babies: the ethical failings and the urgent need for better governance. J Zhejiang Univ-Sci B (Biomed & Biotechnol).

[50] Li L, Rispoli R, Patient R, Ciau Uitz A, Porcher C (2019). Etv6 activates vegfa expression through positive and negative transcriptional regulatory networks in *Xenopus* embryos. Nat Commun.

[51] Lino CA, Harper JC, Carney JP, Timlin JA (2018). Delivering CRISPR: a review of the challenges and approaches. Drug Deliv.

[52] Mali S (2013). Delivery systems for gene therapy. Indian J Human Gene.

[53] Mandip KC, Steer CJ (2019). A new era of gene editing for the treatment of human diseases. Swiss Med Wkly.

[54] Mao Y, Zhang H, Xu N, Zhang B, Gou F, Zhu JK (2013). Application of the CRISPR–Cas system for efficient genome engineering in plants. Mol Plant.

[55] Min Y-L, Bassel-Duby R, Olson EN (2019). CRISPR correction of Duchenne muscular dystrophy. Annu Rev Med.

[56] Mojica FJ, Díez-Villaseñor C, García-Martínez J, Soria E (2005). Intervening sequences of regularly spaced prokaryotic repeats derive from foreign genetic elements. J Mol Evol.

[57] Mooney MR, Davis EE, Nicholas Katsanis N (2019) Analysis of single nucleotide variants in CRISPR-Cas9 edited zebrafish exomes shows no evidence of off-target inflation. Front Genet 11. 10.3389/fgene.2019.00949 10.3389/fgene.2019.00949 PMC6797590 31681410

[58] Mout R, Ray M, Tonga GY, Lee Y-W, Tay T, Sasaki K (2017). Direct cytosolic delivery of CRISPR/Cas9-ribonucleoprotein for efficient gene editing. ACS Nano.

[59] Nakata A, Amemura M, Makino K (1989). Unusual nucleotide arrangement with repeated sequences in the *Escherichia coli* K-12 chromosome. J Bacteriol.

[60] Osborn MJ, Gabriel R, Webber BR, deFeo AP, McElroy AN, Jarjour J (2015). Fanconi anemia gene editing by the cRISPR/cas9 system. Hum Gene Ther.

[61] Park CY, Kim DH, Son JS, Sung JJ, Lee J, Bae S (2015). Functional correction of large factor VIII gene chromosomal inversions in hemophilia a patient-derived iPScs using cRISPR-cas9. Cell Stem Cell.

[62] Park JY, Moon BY, Park JW, Thornton JA, Park YH, Seo KS (2017). Genetic engineering of a temperate phage-based delivery system for CRISPR/Cas9 antimicrobials against *Staphylococcus aureus*. Sci Rep.

[63] Petersen B, Niemann H (2015). Molecular scissors and their application in genetically modified farm animals. Transgenic Res.

[64] Prieto J, Redondo P, López-Méndez B (2018). Understanding the indirect DNA read-out specificity of I-CreI Meganuclease. Sci Rep.

[65] Qi LS, Larson MH, Gilbert LA, Doudna JA, Weissman JS, Arkin AP (2013). Repurposing CRISPR as an RNA-guided platform for sequence-specific control of gene expression. Cell.

[66] Reyes AP, Lanner F (2017). Towards a CRISPR view of early human development: applications, limitations and ethical concerns of genome editing in human embryos. Development.

[67] Richardson CD, Ray GJ, DeWitt MA, Curie GL, Corn JE (2016). Enhancing homology-directed genome editing by catalytically active and inactive CRISPR-Cas9 using asymmetric donor DNA. Nat Biotechnol.

[68] Robb GB (2019). Genome editing with CRISPR-Cas: an overview. Curr Protoc Essent Lab Tech.

[69] Rodrigeuz-Rodrigeuz DR, Ramirez-Solis R, Garza-Elizondo MA, Garza-Rodrigeuz MDL, Barrera-Saldana HA (2019). Genome editing: a perspective on the application of CRISPR/Cas9 to study human diseases (Review). Int J Mol Med.

[70] Rouet P, Smih F, Jasin M (1994). Introduction of double-strand breaks into the genome of mouse cells by expression of a rare-cutting endonuclease. Mol Cell Biol.

[71] Ryu J, Prather RS, Lee K (2018). Use of gene-editing technology to introduce targeted modifications in pigs. J Anim Sci Biotechnol.

[72] Saleh-Gohari N, Helleday T (2004). Conservative homologous recombination preferentially repairs DNA double-strand breaks in the S phase of the cell cycle in human cells. Nucleic Acids Res.

[73] Shen B, Zhang J, Wu H, Wang J, Ma K, Li Z (2013). Generation of gene-modified mice via Cas9/RNA-mediated gene targeting. Cell Res.

[74] Silva G, Poirot L, Galetto R, Smith J, Montoya G, Duchateau P (2011). Meganucleases and other tools for targeted genome engineering: perspectives and challenges for gene therapy. Curr Gene Ther.

[75] Stoddard BL (2014). Homing endonucleases from mobile group I introns: discovery to genome engineering. MobDNA.

[76] Tu Z, Yang W, Yan S, Yin A, Gao J, Liu X (2017). Promoting Cas9 degradation reduces mosaic mutations in non-human primate embryos. Sci Rep.

[77] Vasebi Y, Khakvar R (2014). CRISPR-Cas: the effective immune systems in the prokaryotes. Int J Mol Clin Microbiol.

[78] Walker-Daniels J (2013). CRISPR and genomic engineering. Mater Methods.

[79] Wang H, Yang H (2019). Gene-edited babies: what went wrong and what could go wrong. PLoS Biol.

[80] Wang M, Glass ZA, Xu Q (2017). Non-viral delivery of genome-editing nucleases for gene therapy. Gene Ther.

[81] Wright AV, Nuñez JK, Doudna JA (2016). Biology and applications of CRISPR systems: harnessing nature’s toolbox for genome engineering. Cell.

[82] Wu WH, Tsai YT, Justus S, Cho GY, Sengillo JD, Xu Y (2018). CRISPR repair reveals causative mutation in a preclinical model of retinitis pigmentosa: a brief methodology. Retinal Gene Ther.

[83] Wu X, Kriz AJ, Sharp PA (2014). Target specificity of the CRISPR-Cas9 system. Quant Biol.

[84] Xiao Q, Guo D, Chen S (2019). Application of CRISPR/Cas9-based gene editing in HIV-1/AIDS therapy. Front Cell Infect Microbiol.

[85] Yang H, Wu Z (2018). Genome editing of pigs for agriculture and biomedicine. Front Genet.

[86] Zhang F, Wen Y, Guo X (2014). CRISPR/Cas9 for genome editing: progress, implications and challenges. Hum Mol Genet.

[87] Zhang H, Mccarty N (2016). cRISPR-cas9 technology and its application in haematological disorders. Br J Haematol.

[88] Zhang Y, Zhang F, Li X, Baller JA, Qi Y, Starker CG, Bogdanove AJ, Voytas DF (2013). Transcription activator-like effector nucleases enable efficient plant genome engineering. Plant Physiol.

[89] Zhao D, Li J, Li S, Xin X, Hu M, Price MA, Rosser SJ, Bi C, Zhang X (2020). Glycosylase base editors enable C-to-A and C-to-G base changes. Nat Biotechnol.

